# OP75 Pharmaceutical Innovativeness Index: Evaluation Of Pharmaceutical Innovation In Lung Cancer Treatment Drugs Approved Between 2011 And 2021

**DOI:** 10.1017/S026646232400134X

**Published:** 2025-01-07

**Authors:** Isabela Freitas, Ludmila Gargano, Ariane André, Francisco de Assis Acurcio, Juliana Alvares-Teodoro, Augusto Guerra

## Abstract

**Introduction:**

Lung cancer (LC) ranks second globally in neoplasia diagnoses and exhibits the highest oncological mortality rate. Oncology has seen a marked increase in U.S. Food and Drug Administration (FDA) approvals for novel drugs, notably in the case of LC. This study aims to assess the innovativeness of recently FDA-approved LC treatments compared to existing options, spanning the 2011 to 2021 period.

**Methods:**

The innovativeness of drugs was assessed through the Pharmaceutical Innovativeness Index (PII). The methodology considers population health needs and aggregated clinical benefits, while weighing methodological quality and the adequacy of available evidence. New drugs are analyzed considering available therapeutic alternatives. The assessment assigns an innovativeness score to the drugs, with 1.0 representing the highest degree of innovation. PII was applied to all drugs with their first FDA indication for LC treatment. The evaluation considered specific clinical indications (e.g., treatment of ALK-positive LC), and data from FDA clinical review reports and pivotal clinical trials were considered.

**Results:**

Eighteen new drugs were identified during the period. The drugs were evaluated with a mean PII of 0.615, ranging from 0.474 to 0.811. No discernible trends in innovativeness were observed within specific indications. Overall, the evaluations indicated that these medications were approved to address significant therapeutic needs. However, the added therapeutic value ranged from absent to moderate. Most supporting evidence for these approvals was derived from clinical studies with low risk of bias. Nevertheless, these studies often featured inadequate designs due to the absence of a comparator arm, limiting the assessment of added therapeutic value.

**Conclusions:**

Pharmaceutical innovations in recent years for LC treatment have demonstrated limited clinical benefits when compared to existing alternatives, highlighting a substantial therapeutic need. The application of the PII can assist in healthcare decision-making, guide investments and research efforts, as well as inform pricing and reimbursement of new technologies, thereby contributing to the sustainability of healthcare systems.

